# Social cognition in 22q11.2 deletion syndrome and idiopathic developmental neuropsychiatric disorders

**DOI:** 10.1186/s11689-021-09363-4

**Published:** 2021-04-17

**Authors:** Rhideeta Jalal, Aarti Nair, Amy Lin, Ariel Eckfeld, Leila Kushan, Jamie Zinberg, Katherine H. Karlsgodt, Tyrone D. Cannon, Carrie E. Bearden

**Affiliations:** 1 Department of Psychiatry and Biobehavioral Sciences, University of California, Los Angeles, CA USA; 2 Department of Psychology, Loma Linda University, Loma Linda, CA USA; 3 Neuroscience Interdepartmental Program, University of California, Los Angeles, CA USA; 4 Department of Psychology, University of California, Los Angeles, CA USA; 5 Department of Psychology, Yale University, New Haven, CT USA

**Keywords:** 22q11.2 deletion, Social cognition, Neurocognition, Psychosis, Autism spectrum disorder

## Abstract

**Background:**

22q11.2 deletion syndrome (22q11DS) is a common recurrent neurogenetic condition associated with elevated risk for developmental neuropsychiatric disorders and intellectual disability. Children and adults with 22q11DS often exhibit marked social impairment as well as neurocognitive deficits, and have elevated rates of both autism spectrum disorder (ASD) and psychosis. However, the relationship between the basic processes of social cognition and cognitive ability has not been well studied in 22q11DS. Here, we examined differences in social cognition in 22q11DS, relative to multiple groups of idiopathic neuropsychiatric disorders, and typically developing healthy controls (HC). Additionally, we examined differences in intellectual functioning and its relationship to social cognitive abilities. Finally, we examined the relationship between social cognitive abilities and real-world social behavior.

**Methods:**

We examined social cognition and intellectual functioning in 273 participants (mean age = 17.74 ± 5.18% female = 44.3%): 50 with 22q11DS, 49 youth with first episode psychosis (FEP), 48 at clinical high-risk (CHR) for psychosis, 24 participants with ASD, and 102 HC. Social cognition was assessed using The Awareness of Social Inference Test (TASIT), while reciprocal social behavior was assessed via parent/caregiver ratings on the Social Responsiveness Scale (SRS). Participants were also administered the Wechsler Abbreviated Scale of Intelligence, 2nd edition (WASI-II) to assess intellectual functioning.

**Results:**

The 22q11DS group exhibited significantly lower social cognitive abilities compared to CHR, FEP, and HC groups after controlling for intellectual functioning, but not in comparison to the ASD group. Significant positive correlations were found between social cognition, as measured by the TASIT and IQ across groups. In contrast, no significant relationships were found between TASIT and real-world social behavior (SRS) for any group.

**Conclusions:**

Our findings indicate social cognitive deficits are more prominent in 22q11DS than idiopathic neuropsychiatric conditions across the age range, even after adjusting for global intellectual function. These results contribute to our understanding of the intellectual and social vulnerabilities of 22q11DS in comparison to idiopathic neuropsychiatric disorders. Our findings of robust associations between intellectual ability and social cognition emphasizes the importance of accounting for neurocognitive deficits in social skills interventions and tailoring these existing treatment models for 22q11DS and other populations with intellectual impairment.

**Supplementary Information:**

The online version contains supplementary material available at 10.1186/s11689-021-09363-4.

## Background

22q11.2 deletion syndrome (22q11DS; also known as DiGeorge or Velocardiofacial Syndrome) is caused by a microdeletion of ~ 46 protein-coding genes on the long arm of chromosome 22 [1] and is the most common recurrent contiguous gene deletion syndrome, estimated to occur in about 1 in 4000 live births [2]. It typically results from a de novo deletion, although 10% of cases are familial [3, 4]. 22q11DS is associated with a range of developmental anomalies [5]. The most frequently associated medical comorbidities include congenital heart defects and facial dysmorphology, such as palatal defects, immunodeficiency, velopharyngeal insufficiency, and hypocalcemia [2, 6].

Individuals with 22q11DS often present with developmental delays, as well as broad cognitive impairment [6]. In most individuals with 22q11DS, Full-Scale IQ falls within the intellectually disabled to low average range (*M* = 72.41, SD = + 13.72) [7]. A range of associated cognitive deficits have also been noted in individuals with 22q11DS, particularly difficulties in working memory, attention, executive functioning, visuospatial processing, learning and memory, arithmetic, and sensorimotor abilities [8–15]. These cognitive impairments can often cascade into significant learning difficulties at school, as well as challenges with adaptive life skills including socio-emotional functioning.

In recent years, several studies have characterized deficits in social behavior and social cognition in individuals with 22q11DS. Specifically, these studies have indicated that youth with 22q11DS have difficulties initiating and maintaining social relationships [5, 16]. In the laboratory, individuals with 22q11DS show impairments in identifying facial displays of emotion and a tendency to demonstrate eye-gaze preferences for the mouth over the eyes [9, 17, 18]. Additionally, other studies found that individuals with 22q11DS were significantly less accurate than healthy controls in identifying or matching emotions [1, 9, 19], particularly when identifying fear, anger, and disgust [9, 17]. Furthermore, 22q11DS individuals have demonstrated poorer performance on theory of mind (ToM) tasks wherein they were required to infer the beliefs, attitudes, and intentions of other people [19–21]. However, differences in non-social neurocognitive abilities, such as executive functioning, could in part account for differences in social cognitive performance [5, 15]. Prior studies have also found that impairments in both social cognition and social skills are more pronounced in children and adolescents with 22q11DS than in children with general developmental delays or idiopathic intellectual disability, as well as compared to other genetic conditions such as Williams syndrome [22–24].

In addition, 22q11DS is associated with increased incidence of developmental neuropsychiatric disorders across the lifespan [25]. Based on existing literature, rates of clinical diagnoses within the 22q11DS population are estimated to be 25–50% for autism spectrum disorder [26–28], 30–40% for attention-deficit hyperactivity disorder [29], 20–35% for psychotic disorders, 30–40% for anxiety disorders, and 20–30% for mood disorders [30]. Conversely, about 0.3% of schizophrenia cases in the general population are estimated to carry a 22q11.2 deletion [31].

All of the aforementioned co-morbid neuropsychiatric conditions are also characterized by social cognitive deficits [32]. However, little is known about how these social impairments and broader neurocognitive profiles are the same or different for 22q11DS compared to primary idiopathic developmental neuropsychiatric disorders. One prior study found poorer adaptive social skills in individuals with 22q11DS and psychotic symptoms, compared to 22q11DS youth without psychosis [33]. To the best of our knowledge, however, no published studies to date have investigated differences in social cognition profiles in individuals with primary idiopathic psychotic spectrum conditions or ASD [34, 35] compared to a highly penetrant genetic condition such as 22q11DS. Thus, examining social cognition and its relationship to global intellectual function and social behavior in 22q11DS relative to idiopathic neuropsychiatric disorders can facilitate development of more targeted treatment options, and as a result, improve overall quality of life for this group. Towards this aim, in the current study, we sought to examine patterns of social cognition deficits in a large cohort of individuals with 22q11DS, relative to youth with a recent onset of subclinical psychotic-like symptoms (i.e., a clinical high-risk (CHR) group), youth with first episode psychosis, youth with ASD, and typically developing healthy controls. Specifically, we administered an audiovisual task of social perception that examines one’s ability to interpret another person’s thoughts, intentions, and feelings [36, 37]. A further goal was to examine differences in intellectual functioning among these groups, and its relationship with social cognition performance. Finally, we assessed the relationship of social cognitive task performance to parent/caregiver report of reciprocal social behavior across groups. We hypothesized that (1) individuals with 22q11DS would have significantly poorer social cognition task performance compared to all other clinical groups, even after controlling for intellectual functioning; (2) social cognition would be associated with intellectual abilities across all groups; and (3) social cognitive task performance across groups would be associated with parent/caregiver report of ‘real world’ reciprocal social behavior.

## Methods

### Participants

Our sample consisted of participants aged 12 to 35, enrolled in multiple research studies at the University of California, Los Angeles (UCLA) that used parallel procedures to assess neurocognition and psychopathology. In total, the current analysis includes 273 participants: 50 patients with 22q11.2 deletions (22 males, 28 females), 48 individuals at clinical high risk (CHR) for psychosis (35 males, 13 females), 49 individuals with first-episode psychosis (FEP; 27 males, 22 females), 24 individuals with ASD (20 males, 4 females), and 102 healthy controls (HC; 48 males, 54 females; see Table 1). All participants were recruited via voluntary response sampling techniques through posting of recruitment flyers at various UCLA psychiatric and genetic clinics, as well as our outreach efforts at various public events in the Southern California region. Typically, participants are referred to our research programs (described below) by providers or contact our study staff directly due to our outreach and recruitment efforts including the HC group. Written informed consent was obtained from all participants, and additionally by parents/caregivers for participants under the age of 18, using procedures approved by the Institutional Review Board at UCLA.

**Table 1:** Demographic data for participant groups

	22q11DS (***N*** = 50)	CHR (***N*** = 48)	FEP (***N*** = 49)	ASD (***N*** = 24)	HC (***N*** = 102)	***p*** value
**Mean age, in years (+** **SD)**	17.7 (+ 5.2)	18.1 (+ 3.1)	15.5 (+ 1.6)	15.7 (+ 2.3)	18.6 (+ 3.9)^*^	< .001
**Age range, in years**	12–35	15–27	12–18	12–20	12–28	
**Percent female (*N*)**	56.0% (28 F)	27.1% (13 F)	44.9% (22 F)	16.7% (4 F)^**^	52.9% (54 F)	< .001
**Mean parental education, in years (+** **SD)**	15 (1.30)	12 (1.53)^***^	16 (1.28)	16 (1.1)	15 (1.66)	< .001
**Race/ethnicity**						< .001
**White, Hispanic**	18%	24%	41.4%^***^	26.7%	14.7%	
**White, non-Hispanic**	74%^***^	43%	41.7%	46.6%	27.5%	
**Other**	8%	33%	16.9%	26.7%	57.8%^***^	

*p < 0.05, **p < 0.01, ***p < 0.001

Patients with 22q11DS were recruited from an ongoing UCLA study, with all patients having a molecularly confirmed diagnosis of 22q11.2 deletion syndrome. CHR individuals were recruited from the UCLA Center for the Assessment and Prevention of Prodromal States (CAPPS), a clinical research center that specializes in identifying and treating prodromal adolescents and young adults at high risk for developing psychotic disorders [38–40]. CHR participants met criteria for one of three psychosis risk syndromes, as assessed by the Structured Interview for Prodromal States (SIPS [41];, based on (1) attenuated (sub-threshold) psychotic symptoms; (2) transient, recent-onset psychotic symptoms; or (3) a substantial drop in social/role functioning in conjunction with Schizotypal Personality Disorder diagnosis or presence of psychotic disorder in first-degree relative. FEP individuals were recruited from the UCLA Adolescent Brain-Behavior Research Center (ABBRC). Inclusion criteria for FEP participants involved a diagnosis of psychotic disorder (including schizophrenia, schizophreniform disorder, schizoaffective disorder, and psychotic disorder not otherwise specified), confirmed using the Structured Clinical Interview for DSM-IV Axis I diagnoses (SCID [42];) by trained study personnel. Individuals with ASD were recruited from an ongoing research study at UCLA’s Center for Autism Research and Treatment (CART). Participants were assessed for ASD symptomatology using the Autism Diagnostic Observation Schedule, 2nd edition (ADOS-2 [43];) by trained clinicians. HC were recruited from each of these ongoing studies, with similar inclusion and exclusion criteria: specifically, HC subjects could not have a personal or close family history of any major neurodevelopmental or psychiatric disorder or intellectual disability (IQ < 70). All CHR, FEP, and ASD, and HC participants were verbally screened for known genetic or medical conditions potentially associated with secondary psychosis or ASD [34, 35], and excluded based on known conditions. However, we did not require all our CHR, FEP, ASD, and HC participants to undergo genetic testing to confirm the absence of underlying genetic conditions. Additional exclusion criteria for all groups included significant neurological or medical conditions (not attributable to 22q11.2 deletion), and/or substance use disorder in the past 6 months.

### Cognitive measures

The following assessments were administered to participants within each group across all studies. General intellectual functioning was estimated using the Wechsler Abbreviated Scale of Intelligence, 2nd edition (WASI-II [44];). This included Full-Scale IQ (FSIQ), Verbal IQ (VIQ) estimated using the Vocabulary subtest, and Non-verbal IQ (NVIQ) estimated using the Matrix Reasoning subtest. To assess social cognition, all participants were administered The Awareness of Social Inference Test-Part 3 (TASIT [36, 37];). The TASIT consisted of 16 video-taped vignettes (each lasting between 15 to 60 s) of everyday social situations, assessing one’s ability to draw inferences about the thoughts, intentions, beliefs, and feelings of others engaged in conversational exchanges involving white lies or sarcasm. After viewing each vignette, participants were asked to answer four types of yes or no questions addressing different aspects of communicative intentions of the characters. The first question asked what one character in the scene was doing (Do), the second question asked what the character was trying to say to the other person (Say), the third question asked what the main character was thinking (Think), and the fourth question asked what the main character was feeling (Feel). A TASIT total score was calculated for each participant by adding their responses to each of the 4 questions per scene across all 16 vignettes, ranging from 0 to 64. Additionally, parents/caregivers of 22q11DS, CHR, ASD, and HC participants were administered the Social Responsiveness Scale-2nd Edition (SRS [45];)—a parent/caregiver rating scale to quantify (dimensionally measured) social impairments within the context of the ASD phenotype [46]. The SRS-2 includes the following subscales: Social Awareness, Social Cognition, Social Communication, Social Motivation, and Restricted Interests and Repetitive Behavior. Parents/caregivers of the FEP group were administered the original version of the SRS [47], which included the following subscales: Receptive, Cognitive, Expressive, Motivational Aspects of Social Behavior, and Autistic Preoccupations. The original SRS administered to parents/caregivers of the FEP group was rescored using the updated norms for the revised version (SRS-2), both of which have the same items, so as to be consistent across all groups. The SRS was originally developed to quantify (dimensionally measured) social impairments within the context of the ASD phenotype, but has now been extended to many other clinical populations, including 22q11DS [20, 48], CHR [21, 49, 50], and FEP [21, 49]. It has also been shown to index traits that are continuously distributed in the general population [51]. Both versions of the SRS are 65-item questionnaires utilizing a 4-point Likert scale (0 = not true, 1 = sometimes true, 2 = often true, 3 = almost always true), with a total *T*-score computed across all domains. Both versions of the SRS are identical for ages 4 through 18, although the subscales are labeled differently. There is also considerable overlap between the original SRS and the revised Adult Form for ages 19 and over, despite some items that differ on the second edition [52]. All the participants in this study, including adult participants, lived with the parents/caregivers who completed SRS reports on them.

### Statistical analysis

All statistical analyses were conducted using Statistical Package for the Social Sciences (SPSS) software v. 25 (Armonk, NY, USA). First, we compared demographic variables between groups using independent sample *t* tests for continuous variables such as age and socio-economic status (SES) indexed as mean parental education years, and chi-square tests for categorical variables such as sex and race/ethnicity. Participants significantly differed on age, sex distribution, mean parental education, and race (Table 1); i.e., CHR and HC participants were significantly older, and CHR youth had significantly lower mean parental education than the other groups. There were also proportionally fewer female participants in the ASD group relative to the other groups. Additionally, the 22q11DS group had significantly more participants who identified as white/non-Hispanic, while the FEP group had significantly more participants who identified as being of Hispanic origin compared to all other groups. The HC group had more participants that identified with other racial/ethnicity categories. Hence, these variables were included as covariates in all subsequent group analyses. Second, one-way analysis of covariances (ANCOVAs), in which we adjusted for age, sex, SES, and race/ethnicity, were performed to examine between-group differences in total TASIT score. We followed up on significant overall differences by examining between-group differences in subdomain TASIT scores (Do, Say, Think, and Feel). Following this, exploratory post-hoc analyses were conducted to elucidate directionality of group differences for each significant ANCOVA. Third, we performed ANCOVAs on the WASI-II scores to examine differences in FSIQ, VIQ, and NVIQ between groups. We then performed an ANCOVA to examine group differences in TASIT total performance after additionally controlling for FSIQ scores. To investigate the relationship between IQ and TASIT performance, we conducted Pearson’s correlations between subscores on these measures. Next, as our 22q11DS sample included individuals with comorbid diagnoses of ASD and psychosis, we conducted a follow-up analysis of group differences in TASIT and IQ scores in diagnostic subgroups within our 22q11DS sample: 22q11DS with a comorbid ASD diagnosis (*N* = 17), 22q11DS with a comorbid psychosis diagnosis (*N* = 2), 22q11DS with both psychosis and ASD (*N* = 5), and 22q11DS with no psychosis or ASD diagnosis (*N* = 26). We also performed additional follow-up analysis of group differences in TASIT and IQ scores in idiopathic ASD compared to 22q11DS with a comorbid ASD only diagnosis and 22q11DS with no comorbid ASD or psychosis diagnosis (See Supplementary Material). Finally, we examined between-group differences in parent-report of real-world social behavior, assessed via the SRS total and subscale scores, using one-way ANCOVA, and examined the relationship between TASIT performance and SRS total score using Spearman’s correlations, since the latter scores were not normally distributed. All ANCOVAs and correlational analyses described above were adjusted for multiple comparisons using Bonferroni correction for total number of comparisons. All significance values reported below are Bonferroni adjusted *p* values.

## Results

### Social cognition results

**Fig. 1:**
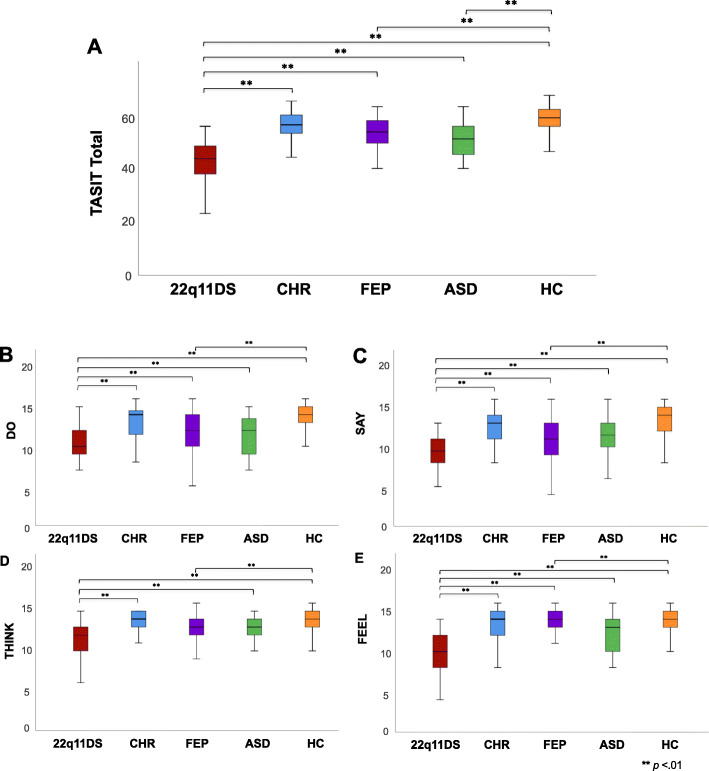
Boxplots showing group differences in TASIT scores. Lower and upper box boundaries indicate 25th and 75th percentiles, respectively, black line inside box represents median, whiskers denote minimum to maximum range, and significant pairwise differences between groups are indicated by asterisks (**=*p* < .01). **a** Group differences for total TASIT score (22q11DS<FEP, ASD, and HC; FEP<HC; and ASD<HC)**. b** Group differences in TASIT score for subdomain Do (22q11DS<CHR, FEP, ASD, and HC; FEP<HC); **c** Group differences in TASIT score for subdomain Say (22q11DS<CHR, FEP, ASD, and HC; FEP<HC). **d** Group differences in TASIT score for subdomain Think (22q11DS<CHR, ASD, and HC; FEP<HC). **e** Group differences in TASIT score for subdomain Feel (22q11DS < CHR, FEP, ASD, and HC; FEP<HC)

Results from the first one-way ANCOVA revealed significant group differences for total TASIT score [*F* (4, 268) = 30.912, *p <* .01]. Post-hoc pairwise comparisons revealed that all groups obtained significantly higher total TASIT scores compared to the 22q11DS group [22q11DS<CHR: (*t*(96) = − 7.991, *p* < .01); 22q11DS<FEP: (*t*(97) = − 3.346, *p* < .01); 22q11DS<ASD: (*t*(72) = − 4.497, *p* < .01); 22q11DS<HC: (*t*(150) = − 12.528, *p* < .01)]. Additionally, FEP and ASD groups obtained significantly lower total TASIT scores than the HC group (Fig. 1a) [HC>FEP: (*t*(149) = 5.459, *p < .*01); HC>ASD: (*t*(124) = 4.306, *p* = .01)]. Follow-up one-way ANCOVAs revealed that the 22q11DS group obtained significantly lower scores across all four subdomains of the TASIT compared to all other groups [Fig. 1b–e]. Additionally, the FEP group obtained significantly lower Do and Say scores than HCs (Fig. 1b, c). The 22q11DS group obtained significantly lower Think scores than CHR, ASD, and HC groups, but did not differ from the FEP group, who in turn performed more poorly than the HC group (Fig. 1d). Additionally, the FEP group obtained significantly lower Feel scores than the HCs (Fig. 1e). See Table 2 for means and standard deviations on TASIT total and subscores for each group.

**Table 2:** Group means and standard deviations for TASIT (raw scores)

	22q11DS Mean (SD)	CHR Mean (SD)	FEP Mean (SD)	ASD Mean (SD)	HC Mean (SD)
Do	10.36 (2.00)^a^	13.06 (2.09)	11.69 (2.71)^b^	11.67 (2.58)	13.56 (1.91)
Say	9.50 (2.06)^a^	12.31 (2.30)	10.88 (3.05)^b^	11.33 (2.63)	13.24 (2.02)
Think	11.48 (2.03)^a^	13.44 (1.38)	12.33 (2.81)^b^	13.25 (1.26)	13.87 (1.23)
Feel	9.90 (2.42)^a^	13.19 (2.21)	12.22 (2.97) ^b^	12.33 (2.18)	13.67 (1.79)
Total	41.24 (6.64)^a^	52.74 (5.74)	49.36 (7.36)^b^	48.58 (6.45)^c^	54.55 (5.75)

^a^22q11DS<CHR, FEP, ASD, HC

^b^FEP<HC

^c^ASD<HC

### Intellectual functioning (IQ) results

**Fig. 2:**
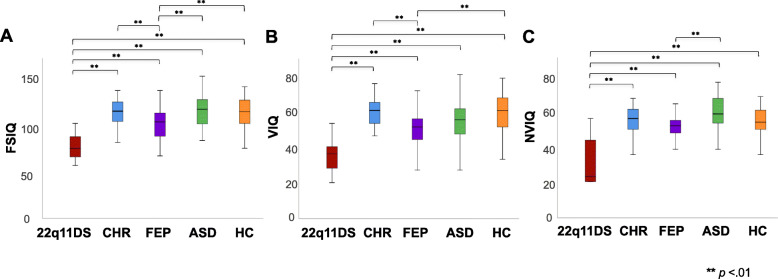
Boxplots showing group differences in FSIQ, VIQ, and NVIQ scores. Lower and upper box boundaries indicate 25th and 75th percentiles respectively, black line inside box represents median, whiskers denote minimum to maximum range, and significant pairwise differences between groups are indicated by asterisks (**=*p* < .01). **a** Group differences in FSIQ (22q11DS<CHR, FEP, ASD, and HC; FEP<CHR, ASD, and HC)**. b** Group differences in VIQ score (22q11DS<CHR, FEP, ASD, and HC; FEP<CHR and HC). **c** Group differences in NVIQ score (22q11DS<CHR, FEP, ASD, and HC; FEP<ASD)

Next, one-way ANCOVAs of WASI-II FSIQ scores revealed a significant overall main effect, indicating significant group differences in FSIQ [Fig. 2; *F* (4, 265) = 52.106, *p* < .01]. Post-hoc tests revealed that 22q11DS individuals obtained significantly lower FSIQ scores compared to all other groups [Fig. 2a], and the FEP group obtained significantly lower FSIQ scores than both CHR, ASD, and HC groups. Significant group differences were also observed for VIQ [*F* (4, 265) = 46.106, *p* < .01], with 22q11DS individuals obtaining significantly lower VIQ scores compared to all other groups (Fig. 2b). Additionally, the FEP group obtained significantly lower VIQ scores than both CHR and HC groups (*p* < .01 for both comparisons), but did not differ from the ASD group (*p* = .50, n.s.). Similarly, 22q11DS individuals obtained significantly lower NVIQ scores than all other groups [*F* (4, 233) = 45.635, *p* < .01; Fig. 2c]. Additionally, youth with FEP obtained significantly lower NVIQ scores than those with ASD (Fig. 2c), but did not significantly differ from HC (*p =* .12, n.s.). See Table 3 for means and standard deviations of WASI-II IQ scores for each group.

**Table 3:** Group means and standard deviations for WASI-II IQ scores

	22q11DS Mean (SD)	CHR Mean (SD)	FEP Mean (SD)	ASD Mean (SD)	HC Mean (SD)
FSIQ	74.49 (13.89)^a^	108.58 (15.62)	98.48 (15.49)^b, c, d^	110.17 (16.41)	110.40 (15.27)
Verbal IQ (*T*-score)	34.61 (9.39)^a^	57.27 (10.23)	49.38 (10.91)^b, c^	54.58 (11.84)	58.26 (10.78)
Non-verbal IQ (*T*-score)	30.92 (12.55)^a^	52.06 (10.30)	47.89 (10.37)^d^	58.50 (8.83)	52.83 (8.87)

^a^22q11DS<CHR, FEP, ASD, HC

^b^FEP<HC

^c^FEP<CHR

^d^FEP<ASD

### Social cognition performance controlling for IQ

**Fig. 3:**
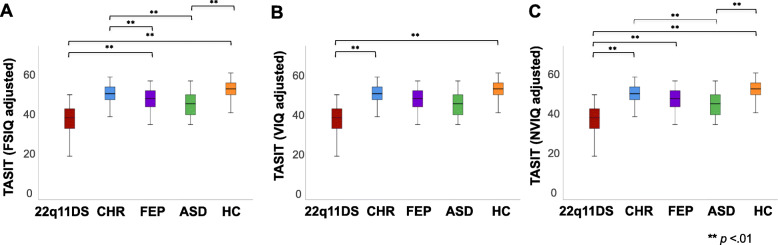
Boxplots showing group differences in TASIT total score after covarying for FSIQ. Lower and upper box boundaries indicate 25th and 75th percentiles respectively, black line inside box represents median, whiskers denote minimum to maximum range, and significant pairwise differences between groups are indicated by asterisks (**=*p* < .01). **a** Group differences in TASIT total score after covarying for WASI-II FSIQ score (22q11DS<FEP and HC; FEP<CHR; ASD<CHR and HC). **b** Group differences in TASIT total score after covarying for WASI-II VIQ (22q11DS<CHR and HC). **c** Group differences in TASIT total score after covarying for WASI-II NVIQ (22q11DS<CHR, FEP, and HC; ASD<CHR and HC)

A follow-up univariate ANCOVA revealed that significant group differences in TASIT performance remained after controlling for IQ [Fig. 3; *F* (4, 264) = 7.471, *p* < .01]. Specifically, after controlling for FSIQ, 22q11DS participants still had significantly poorer performance on the TASIT (total score) relative to FEP and HC, but did not differ from CHR (*p* = .66, n.s.) and ASD (*p* = .89, n.s.; Fig. 3a). Additionally, controlling for FSIQ, FEP participants still had significantly lower TASIT total scores than CHR, and ASD participants had significantly lower TASIT total scores than CHR and HC (Fig. 3a). Next, we wanted to determine whether controlling for VIQ or NVIQ specifically impacted TASIT performance across groups. After controlling for VIQ, significant group differences in TASIT performance remained [*F* (4, 264) = 6.119, *p* < .01], with 22q11DS individuals obtaining significantly lower TASIT total scores than CHR and HC groups, but did not differ from FEP (*p* = .48, n.s) and ASD group (*p* = .61, n.s.; Fig. 3b). Additionally, significant between-group differences in TASIT performance was also observed after controlling for NVIQ [*F* (4, 232) = 10.099, *p* < .01], with 22q11DS individuals obtaining significantly lower TASIT total scores than CHR, FEP, and HC groups, but not compared to the ASD group (*p* = .72, n.s.). The ASD group also obtained significantly lower TASIT total scores than both CHR and HC groups, controlling for NVIQ (Fig. 3c).

### Relationship between social cognition performance and IQ

**Fig. 4:**
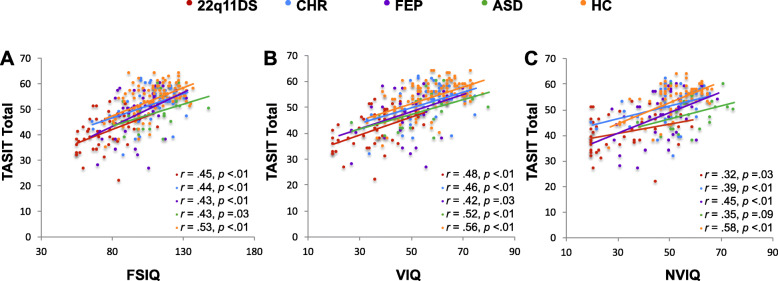
**a** Scatterplot showing correlation between TASIT and FSIQ for 22q11DS, CHR, FEP, ASD, and HC. IQ is positively and significantly correlated with TASIT total score for all groups. **b** Scatterplot showing correlation between TASIT total score and VIQ across groups. VIQ score is positively and significantly correlated with TASIT total score for all groups. **c** Scatterplot showing correlation between TASIT total score and NVIQ across groups. NVIQ score is positively and significantly correlated with TASIT total score for 22q11DS, CHR, FEP, and HC groups (but not ASD)

Pearson’s correlation results indicated positive relationships between TASIT total score and FSIQ as well as VIQ for all groups, such that higher intellectual functioning was associated with better TASIT performance in each group (Fig. 4a, b). Lastly, we found significant positive correlations between TASIT and NVIQ for all groups, except ASD (Fig. 4c).

### Reciprocal social behavior

**Fig. 5:**
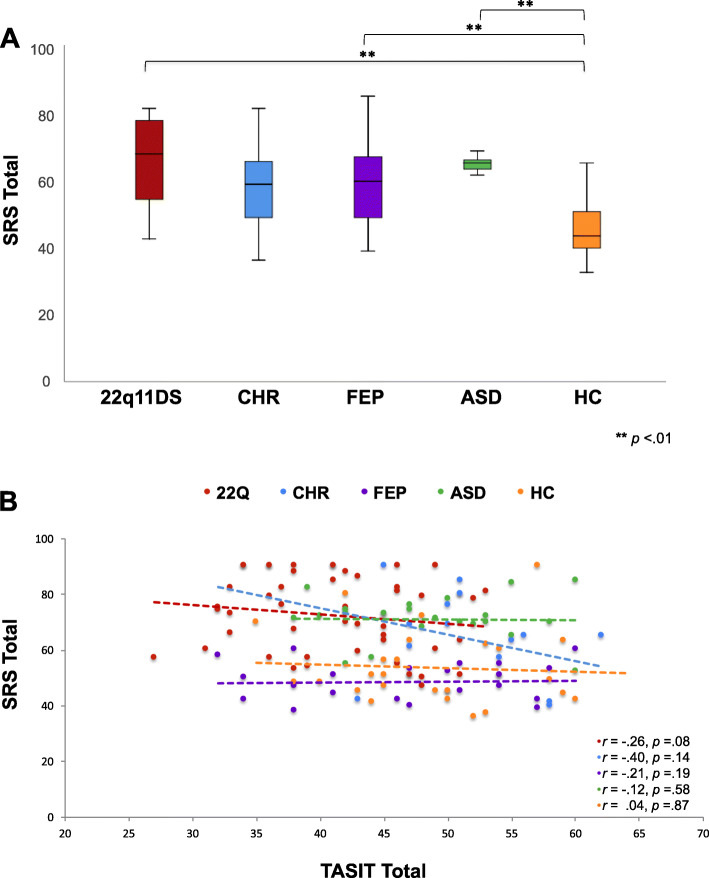
**a** Boxplots showing group differences in SRS total score. Lower and upper box boundaries indicate 25th and 75th percentiles, respectively, black line inside box represents median, whiskers denote minimum to maximum range, and significant pairwise differences between groups are indicated by asterisks (**=*p* < .01) (HC<22q11DS, FEP, and ASD). **b** Scatterplot showing correlations between TASIT total score and SRS by group: no significant correlations found

Results from the one-way ANCOVA revealed significant group differences in SRS total score across groups [Fig. 5a; *F* (4, 140) = 9,861 *p* < .01]; HC participants obtained significantly lower SRS total scores compared to all other groups, indicating more normative social behavior (*p* < .01, for all comparisons), except CHR (*p* = .07, n.s.). While clinical groups (22q11DS, CHR, FEP, and ASD) obtained clinically elevated mean SRS total scores, there were no significant differences in SRS score between clinical groups (Supplementary Table 1). Lastly, we examined the relationship between TASIT performance and real-world social behavior, as assessed via the SRS, for each group. However, no correlations between these measures survived correction for multiple comparisons (Fig. 5b). We also ran an exploratory analysis examining the association between SRS subscale scores and TASIT total score for each group, and did not find any associations between subscale scores and TASIT performance that survived correction for multiple comparisons (Supplementary Table 2).

### 22q11DS subgroup analyses

Within the 22q11DS sample, we found significant differences in VIQ [*F* (3, 45) = 4.743, *p* = .01], and relatedly FSIQ [*F* (3, 45) = 3.397, *p* = .03], as a function of psychiatric diagnosis, but no differences in NVIQ (*p* = .29, n.s.). Specifically, 22q11DS patients with both psychosis and ASD obtained significantly lower VIQ (and FSIQ) than the 22q11DS group with no comorbid diagnosis. Intellectual functioning (FSIQ, VIQ, and NVIQ) did not differ between 22q11DS group with no comorbid diagnoses compared to those with psychosis-only diagnosis, nor those with ASD-only diagnosis (*p* > 0.9, n.s., for all comparisons). No significant differences in TASIT total score [*F* (3, 46) = 1.890, *p =* .15] and SRS total score [*F* (3, 42) = 2.894, *p* = .09] were observed as a function of ASD/psychosis comorbidity in 22q11DS. See Table 4 for means and standard deviations of TASIT, WASI, and SRS scores for each subgroup.

**Table 4:** 22q11DS subgroup means and standard deviations for TASIT, SRS, and WASI-II IQ scores

	22q11DS with no ASD or psychosis diagnosis	22q11DS with ASD	22q11DS with psychosis	22q11DS with both ASD and psychosis
	(***N*** = 26, 52%)	(***N*** = 17, 34%)	(***N*** = 2, 4%)	(***N*** = 5, 10%)
	Mean (SD)	Mean (SD)	Mean (SD)	Mean (SD)
TASIT total score	42.92 (5.78)	40.59 (7.94)	35.50 (3.54)	37.00 (4.00)
FSIQ	77.20 (13.52)	76.18 (13.19)	68.00 (18.39)	57.80 (3.11)^a^
Verbal IQ	37.04 (8.35)	35.41 (9.17)	29.00 (12.73)	22.00 (2.92)^a^
Non-verbal IQ	31.92 (13.64)	32.71 (12.09)	28.50 (12.02)	20.80 (0.84)
SRS total score	67.26 (15.03)	77.31 (10.77)	81.50 (12.02)	80.80 (10.16)

^a^22q11DS with both ASD and psychosis<22q11DS with no ASD or psychosis diagnosis

## Discussion

To the best of our knowledge, this is the first study to directly examine the relationship between basic processes of social cognition, intellectual ability, and social behavior in individuals with 22q11DS compared to multiple primary idiopathic neuropsychiatric disorders simultaneously. We found that individuals with 22q11DS have more impaired social cognition than individuals with CHR and FEP, as well as healthy controls. While social cognition deficits in 22q11DS have been well established [9, 20, 21, 53], our results further illustrate that these deficits are more prominent than those observed for CHR and FEP groups across the age range, even after adjusting for global intellectual function. Additionally, despite group differences in overall intellectual functioning, 22q11DS individuals did not demonstrate significant social cognition deficits compared to the ASD group after adjusting for global intellectual function. This suggests that the social impairments present within this syndrome are not wholly accounted for by broad deficits in cognitive functioning. 22q11DS individuals both with and without comorbid psychiatric conditions consistently scored lower across all subdomains of the TASIT, highlighting their deficits in understanding reciprocal social exchange, and picking up lies and sarcasm in tone of voice, facial expression, and other visual or conversational cues. It is likely that haploinsufficiency of genes within the 22q11.2 locus and associated emotional and behavioral challenges [25] contribute to the observed substantial social cognition deficits in this population [53].

Consistent with prior work [17, 54, 55], we also found that individuals with 22q11DS had lower overall intellectual functioning across both verbal and non-verbal domains in comparison to heathy controls, as well as idiopathic ASD. Our findings additionally indicate that 22q11DS patients are impaired on overall intellectual functioning relative to idiopathic CHR and FEP. Regardless of the range of intellectual functioning, however, higher IQ was associated with better social cognition performance across all groups.

Although 22q11DS participants consistently showed poorer social cognition relative to CHR, FEP, and healthy controls, impairments were also present in youth with FEP (although this effect was no longer significant after controlling for IQ). This is in contrast to CHR youth, who did not show deficits in social cognition performance compared to HC. This finding, albeit cross-sectional, suggests there may be a decline in social cognition from prodrome to onset of overt psychotic symptoms. Prior studies have revealed that adult patients with psychosis show significant social cognitive deficits, particularly in theory of mind and social perception domains [56–59]. We also found poorer social cognition performance in participants with ASD relative to HC participants, consistent with the core social interaction deficits characteristic of this group [60]. Additional comparisons indicated that social cognition performance was better in participants with idiopathic ASD relative to 22q11DS subgroups with and without comorbid ASD. This suggests that even though behaviorally defined neuropsychiatric disorders are characterized by poorer social cognition performance compared to healthy controls, individuals with 22q11DS (both with and without comorbid neuropsychiatric disorders) experience greater impairments in social cognition compared to individuals with these idiopathic conditions.

Apart from the role of broader cognitive deficits accounted for in the present study, there are several more nuanced aspects of neurocognition that play a crucial role in social cognition abilities such as working memory and executive functioning skills [61–65]. Specifically, working memory is crucial for the encoding and retention of important social cues such as facial expression that are necessary to navigate the social world adequately [63, 64]. Additionally, working memory deficits have been associated with poor overall social competence and peer rejection in youth [66]. Working memory has also been strongly associated with the development of theory of mind skills through childhood and adolescence [67], and would hence be expected to influence performance on social cognition measures such as the TASIT. While working memory deficits are of particular concern in 22q11DS [5, 68–70], impairments in this domain are often seen in CHR [71–73], FEP [74–76], and ASD [77–79] as well. Given these findings, the working memory deficits characteristic of each of these neuropsychiatric conditions may play a role in the deficits in social cognition performance observed. Since working memory skills and fluid intelligence are highly correlated [80–83], it is possible that the observed relationships between broader intellectual functioning and social cognition performance across our patient groups are mediated by deficits in the working memory domain. Hence, further research into the specific role of working memory abilities in social cognition performance across these developmental neuropsychiatric disorders is warranted.

Finally, parent/caregiver report of real-world social behavior revealed considerable variability across all clinical groups, but were within a more concentrated range for the ASD group. On average, however, parent/caregiver reports of social difficulties fell within the clinically elevated range across all patient groups. Even within the 22q11DS subgroups, parent/caregiver reports of social difficulties were clinically elevated for all subgroups with or without comorbidities. Despite this, we did not find a significant relationship between social cognition performance and real-world social behavior for any clinical group, although the findings were trending in the expected direction across all groups. While numerous studies have used ToM tasks and the SRS concurrently in order to broadly examine social outcomes in our specified clinical groups, few have directly assessed the relationship between the two types of measures [20, 48, 84]. A recent study in ASD adolescents found that combined poor performance on several social cognition tasks (ToM tasks, emotion-recognition tasks, and anthropomorphic animations) was collectively associated with elevated SRS scores [85]. Some ASD studies have further found that parent/caregiver report of real-world social behaviors are better measures of treatment-related changes in social outcomes than are social cognition tasks, which suggests that these tasks may be measuring more nuanced social cognition skills such as ToM or attribution of intentions compared to everyday reciprocal social behaviors assessed by the SRS [86, 87]. One study on 22q11DS found a significant inverse association, indicating poorer performance on a ToM task (anthropomorphic animations) was associated with higher SRS scores [20]. Specific to the relationship between TASIT and SRS, a prior study did not find a significant association between TASIT performance and SRS in 22q11DS [48] in line with our findings. One study in adults meeting CHR criteria found a significant association between higher TASIT scores and lower self-reported SRS scores [50]. Another study on adults with ASD found a significant association between higher TASIT scores and higher self-reported SRS scores [88]. Hence, findings in the literature of the association between TASIT and SRS seem mixed, and likely vary as a result of primary diagnosis, self- versus other-report, and maybe insight into the participants’ own social difficulties with increasing age. Several studies that have specifically examined the relationship between performance-based measures and parent/caregiver reports of attention, executive functioning, memory, internalizing, and externalizing behaviors in a variety of neurodevelopmental conditions have found limited associations between the two types of measure suggesting that laboratory-based performance measures and parent report may be assessing different aspects of the same cognitive domain [89–93]. Hence, it is possible that the types of social concerns endorsed on the SRS (e.g., eye contact, odd behaviors, preference for being alone, ritualistic behaviors) may not relate as closely as we originally hypothesized to the TASIT’s measurement of nuanced social cognition skills such as lie and sarcasm detection even though they are both measures of social functioning.

In sum, our findings emphasize further need for assessment and interventions focused on improvement of social perception and social functioning in individuals with 22q11DS. Due to the overt medical problems that require urgent attention, intervention for social skills is more frequently overlooked in 22q11DS. In a recent review of previous cross-sectional as well as longitudinal studies assessing the early clinical signs in 22q11DS in predicting psychosis co-morbidity [94], it was collectively found that poor social functioning was linked to elevated negative and positive symptoms of psychosis. It may be beneficial for individuals with 22q11DS to be routinely referred by clinicians for social and cognitive skills training. Evidence from previous studies in idiopathic psychosis [95] and ASD [96] suggests that psychosocial and social skills training show positive treatment effects for both groups even 3–4 months post-treatment on average, suggesting good generalizability of social skills treatment gains, and improved overall functioning and sense of well-being. Intervention models directed at improving psychosocial function in individuals with 22q11DS have previously been proposed, with one pilot study that found targeted training in the identification of non-verbal social cues and perspective taking is associated with significant gains in social cognitive abilities in adolescents with 22q11DS [97]. Adapting existing treatment models from idiopathic ASD and psychosis to better suit the cognitive needs of individuals with 22q11DS could improve social cognition, and thereby overall quality of life. We further suggest close monitoring and early social skills intervention for individuals at risk for psychosis (whether genetically or behaviorally defined), before they begin to experience the decline in functioning related to onset of psychotic symptoms. Our findings of an association between intellectual ability and social functioning emphasizes the importance of accounting for neurocognitive deficits in these social skills interventions and tailoring these existing treatment models for both the 22q11DS and FEP populations. Our findings also suggest involving parents/caregivers more actively in social skills intervention for these groups, as their reported concerns in this domain may be valuable information that is not necessarily always captured by our existing assessments of social cognition.

## Limitations and conclusions

The main limitation of this study is that our data were collected cross-sectionally across different research projects pertaining to each clinical population, and thus we were only able to compare those measures available across all clinical groups (WASI-II, TASIT, SRS). Further research is needed to examine other aspects of neurocognition, such as working memory, that may influence social skills beyond broad intellectual abilities, as well as inclusion of more comprehensive measures of social cognition. Additionally, SRS data were missing for several participants across groups, which could have impacted the lack of association with TASIT performance in our study. Lastly, our ASD sample was small (*N* = 24), as this is part of a newer ongoing study (K99MH113820; Author AN). Hence, findings for the ASD group may be underpowered compared to the other groups.

In conclusion, our findings suggest that social impairments are associated with neurocognitive deficits across all clinical groups, but more pronounced in 22q11DS compared to idiopathic neuropsychiatric disorders. These results contribute to the understanding of the intellectual and social vulnerabilities of 22q11DS in comparison to idiopathic neuropsychiatric disorders. The inclusion of multiple comparison groups with these conditions was essential to explore the specificity of social cognition deficits in 22q11DS. Social cognition deficits—and their relationship to intellectual functioning—are important factors in the clinical management of individuals with 22q11DS. Development of targeted interventions tailored to address social and intellectual difficulties experienced by 22q11DS is crucial to preventing declines in overall functioning and improving quality of life.

## Supplementary Information

**Additional file 1: Supplementary Table 1**. Group means and standard deviations for SRS total and subscale T-scores. **Supplementary Table 2**. Spearman correlations between TASIT total score and SRS subscale scores. **Supplementary Table 3**. ASD and 22q11DS subgroup means and standard deviations for TASIT, SRS, and WASI-II IQ scores

## Data Availability

The data supporting the conclusions of this article are included within the article and its additional files.

## Abbreviations

22q11DS: 22q11.2 deletion syndrome
CHR: Clinical high-risk
FEP: First-episode psychosis
ASD: Autism spectrum disorder
HC: Healthy controls
TASIT: The Awareness of Social Inference Test
SRS: Social Responsiveness Scale
IQ: Intellectual functioning
WASI-II: Wechsler Abbreviated Scale of Intelligence-Second Edition
ToM: Theory of mind
UCLA: University of California, Los Angeles
CAPPS: Center for the Assessment and Prevention of Prodromal States
SIPS: Structured Interview for Prodromal States
ABBRC: Adolescent Brain-Behavior Research Center
CART: Center for Autism Research and Treatment
ADOS-2: Autism Diagnostic Observation Schedule-Second Edition
FSIQ: Full-Scale IQ
VIQ: Verbal IQ
NVIQ: Non-verbal IQ
SPSS: Statistical Package for the Social Sciences
SES: Socio-economic status
ANOVAs: Analysis of variance
ANCOVA: Analysis of covariance
