# Supplementary material for: Social cognition in 22q11.2 deletion syndrome and idiopathic developmental neuropsychiatric disorders
Source: J Neurodev Disord. 2021 Apr 17;13:15. doi: 10.1186/s11689-021-09363-4 (PMC8052741; doi:10.1186/s11689-021-09363-4)
Supplement: Supplementary file 1 — Additional file 1: Supplementary Table 1. Group means and standard deviations for SRS total and subscale T-scores. Supplementary Table 2. Spearman correlations between TASIT total score and SRS subscale scores. Supplementary Table 3. ASD and 22q11DS subgroup means and standard deviations for TASIT, SRS, and WASI-II IQ scores [file 11689_2021_9363_MOESM1_ESM.docx]

**Supplementary Material**

**Secondary Analyses: 22q11DS Subgroups**

Results of the secondary analysis comparing 22q11DS subgroups (i.e., with no comorbid diagnoses, 22q11DS with ASD-only, 22q11DS with both ASD and psychosis, and idiopathic ASD) revealed significant group differences in TASIT performance and intellectual functioning, but no significant differences in SRS (Supplementary Table 3). Specifically, the idiopathic ASD group obtained significantly higher TASIT total scores [*F* (3, 68) = 7.721, *p*=.01]; VIQ [*F* (3, 67) = 25.385, *p*=.01]; NVIQ [*F* (3, 67) = 32.694, *p*=.01]; and FSIQ [*F* (3, 67) = 35.884, *p*=.01] than 22q11DS with ASD-only diagnosis, 22q11DS with both ASD and psychosis, and 22q11DS with no comorbid diagnoses. However, no significant differences in SRS total score were observed between the 22q11DS subgroups and idiopathic ASD [*F* (3, 63) = 3.368, *p*=.12, n.s.].

**Supplementary Table 1:** Group means and standard deviations for SRS total and subscale T-scores

|  | **22q11DS**  **N=46**  **Mean (SD)** | **CHR**  **N=15**  **Mean (SD)** | **ASD**  **N=23**  **Mean (SD)** | **HC**  **N=22**  **Mean (SD)** |  | **FEP**  **N=41**  **Mean (SD)** |
| --- | --- | --- | --- | --- | --- | --- |
| Social Awareness | 61.63 (15.67) | 59.67 (12.12) | 69.74 (9.34) | 51.09 (13.79)^a^ | Receptive | 57.17 (12.50) |
| Social Cognition | 73.37 (13.58) | 60.53 (14.50) | 65.48 (7.67) | 52.18 (13.27)^a^ | Cognitive | 63.83 (15.15) |
| Social Communication | 70.76 (14.35) | 61.27 (16.20) | 70.61 (9.02) | 51.45 (12.65)^a^ | Expressive | 63.61 (13.44) |
| Social Motivation | 68.67 (14.86) | 60.53 (17.50) | 68.26 (13.90) | 53.27 (12.00)^a^ | Motivational Aspects of Social Behavior | 66.76 (12.57) |
| Restricted Interests and Repetitive Behaviors | 72.59 (14.00) | 65.33 (15.78) | 69.09 (10.42) | 50.18 (12.14)^a^ | Autistic Preoccupations | 64.37 (14.35) |
| SRS-2 Total | 72.85 (13.96) | 63.60 (15.55) | 71.04 (8.14) | 51.64 (12.66)^a^ | SRS Total | 65.22 (13.37) |

a = HC < 22q11DS, ASD, FEP

**Supplementary Table 2:** Spearman correlations between TASIT total score and SRS subscale scores

| **TASIT Total Score** | **SRS Social Awareness** | **SRS Social Cognition** | **SRS Social Communication** | **SRS Social Motivation** | **SRS Restricted Interests and Repetitive Behaviors** |
| --- | --- | --- | --- | --- | --- |
| 22q11DS | -0.116 | -0.318 | -0.248 | -0.059 | -0.144 |
| CHR | -0.407 | -0.112 | -0.409 | -0.272 | -0.231 |
| FEP | -0.283 | -0.236 | -0.231 | -0.062 | -0.199 |
| ASD | -0.244 | -0.322 | 0.047 | 0.088 | 0.092 |
| HC | 0.051 | -0.052 | 0.187 | 0.004 | 0.165 |

**Supplementary Table 3:** ASD and 22q11DS subgroup means and standard deviations for TASIT, SRS, and WASI-II IQ scores

|  | **Idiopathic ASD**  **(N=24)**  **Mean (SD)** | **22q11DS with**  **ASD**  **(N=17)**  **Mean (SD)** | **22q11DS with both ASD and psychosis**  **(N=5)**  **Mean (SD)** | **22q11DS with no ASD or psychosis diagnosis**  **(N=26)**  **Mean (SD)** |
| --- | --- | --- | --- | --- |
| TASIT Total Score | 48.58 (6.45)^a^ | 40.59 (7.94) | 37.00 (4.00) | 42.92 (5.78) |
| FSIQ | 110.17 (16.41)^a^ | 76.18 (13.19) | 57.80 (3.11) | 77.20 (13.52) |
| Verbal IQ | 54.58 (11.84)^a^ | 35.41 (9.17) | 22.00 (2.92) | 37.04 (8.35) |
| Nonverbal IQ | 58.50 (8.83)^a^ | 32.71 (12.09) | 20.80 (0.84) | 31.92 (13.64) |
| SRS Total Score | 71.04 (8.14) | 77.31 (10.77) | 80.80 (10.16) | 67.26 (15.03) |

a = Idiopathic ASD > 22q11DS with ASD, 22q11DS with both ASD and psychosis, and 22q11DS with no ASD or psychosis diagnosis
